# Progress in photocatalytic degradation of industrial organic dye by utilising the silver doped titanium dioxide nanocomposite

**DOI:** 10.1016/j.heliyon.2024.e40998

**Published:** 2024-12-05

**Authors:** Nirosha Ramesh, Chin Wei Lai, Mohd Rafie Bin Johan, Seyyed Mojtaba Mousavi, Irfan Anjum Badruddin, Amit Kumar, Gaurav Sharma, Femiana Gapsari

**Affiliations:** a Nanotechnology and Catalysis Research Centre (NANOCAT), Institute for Advanced Studies (IAS), University of Malaya (UM), 50603, Kuala Lumpur, Malaysia; b Mechanical Engineering Department, Faculty of Engineering, Brawijaya University, MT Haryono167, Malang, 65145, Indonesia; c Department of Chemical Engineering, National Taiwan University of Science and Technology, Taiwan; d Department of Allied Sciences, School of Health Sciences and Technology, UPES, Dehradun, 248 007, India; e International Research Centre of Nanotechnology for Himalayan Sustainability (IRCNHS), Shoolini University, Solan, 173229, India

**Keywords:** Silver, Titanium dioxide, Nanocomposite, Photocatalytic degradation, Organic dye

## Abstract

Industrial organic dyes represent a significant portion of pollutants discharged into the environment, particularly by the textile industry. These compounds pose serious threats to living organisms due to their high toxicity. Various techniques have been explored for the degradation of organic dyes, among which heterogeneous photocatalysis utilising titanium dioxide (TiO_2_) stands out as a promising technology. However, the practical application of TiO_2_ as photocatalyst has limitations for the following reasons; First, TiO_2_ has a low sensitivity to visible light due to a large band gap which can be 3.2 eV for the anatase polymorph. Second, the recombination rate of photo-induced electron-hole pairs in TiO_2_ is very fast. Recent research studies have brought to light that a silver-doped titanium dioxide nanocomposite could be one of the promising answers to these problems. This nanocomposite has garnered significant attention because of its unique features that suggest the manifestation of more effective concepts to minimize the electron-hole recombination and broaden light absorption. This causes Schottky barrier which is essentially created by integrating the silver nanoparticles into titanium dioxide. It is quite significant in decelerating the recombination of the electron-hole pairs, thus increasing photocatalytic activity. Further, it is more effective in that the use of silver also widens the titanium dioxide absorption range to the visible light hence maximizing capture and conversion of broader range of light energies for catalytic reactions.

This paper therefore seeks to examine the research background regarding the industrial organic dyes starting with the history of industrial organic dyes before delving into an evaluation of the current and most current research on industrial organic dyes looking at advanced methods of their degradation with specific focus on silver-doped TiO_2_ for photocatalytic enhancement. This paper also reviews the experimental work concerning the actual photocatalytic degradation process and presents the factors affecting the performance of silver-doped TiO_2_ nanocomposites by eliminating organic dyes from wastewater. It also encompasses a general background into the various synthesis methods used in the preparation of silver-doped TiO_2_ nanocomposites. Additionally, challenges and future perspectives in the field are outlined, with a focus on the development of novel strategies to further improve the efficiency and sustainability of silver-doped TiO_2_ photocatalysts for industrial organic dye degradation. In conclusion, this review offers a significant outlook on the existing literature concerning the silver-doped TiO_2_ nanocomposites for effective photocatalytic degradation of the industrial organic dyes because of the rising pollution level and helping future researchers in seeking the solutions for environmental issues and developing sustainable wastewater treatment.

## Graphical abstract

**Figure:**
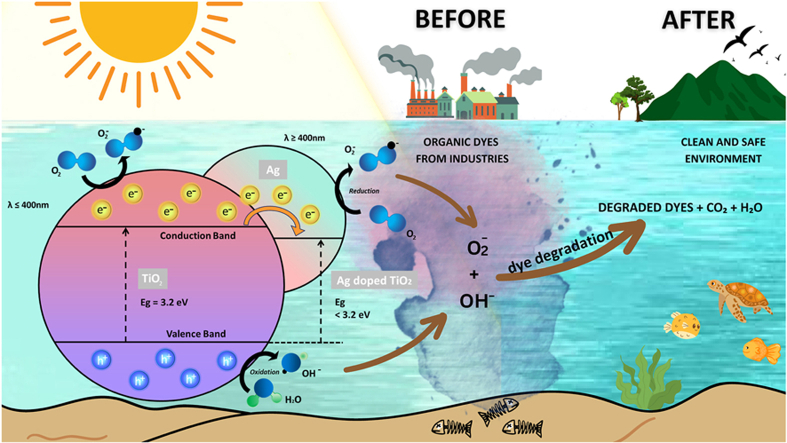

## 1 Introduction

United Nations International Children's Fund (UNICEF) and the World Health Organization (WHO) report that nearly one-third of the global population lacks access to clean water, with an estimated 1.5 million annual deaths resulting from waterborne diseases [1]. According to the United Nations World Water Development Report, over 80 % of wastewater from various sectors is released into the environment without adequate treatment [2]. Among industrial polluters, textile dyeing is one of the largest, accounting for 20 % of global wastewater production [3,4]. Specifically, the textile industry contributes significantly to water pollution by releasing highly toxic and non-biodegradable dyes, which severely threaten ecosystems [5]. These dyes impede light penetration in water, disrupting photosynthesis in aquatic plants and organisms [6]. Furthermore, synthetic dyes have been linked to adverse health effects, including respiratory issues, skin sensitization, and carcinogenicity [7].

Given these environmental and public health challenges, there is a pressing need for cost-effective, eco-friendly methods to clean water sources contaminated with organic pollutants. This pollution not only threatens ecosystems but also undermines efforts to achieve the United Nations Sustainable Development Goal (UNSDG) 6, which seeks to ensure clean water and sanitation for all. Addressing industrial pollution, particularly through textile dye remediation, is crucial to achieving this goal and protecting public health. Advanced Oxidation Processes (AOPs) such as ozonation, catalytic wet peroxide reaction (CWPO), and photocatalysis, align with Sustainable Development Goal (SDG) 6 by providing eco-friendly solutions to remediate contaminated water sources. These technologies advance sustainable environmental practices by generating reactive oxygen species that degrade pollutants [8], contributing to cleaner water systems in line with the UNSDG's clean water initiative [9]. CWPO, for instance, has proven effective in degrading organic pollutants like phenol through catalysts such as Cu(II)- impregnated zeolites and Co(II)-pyrophyllite [[10], [11], [12]]. Among these processes, photocatalysis has emerged as a green solution for tackling pollution. Photocatalysis utilizes photocatalysts to produce reactive oxygen species, including hydroxyl radicals (•OH) and superoxide radicals (O₂•−), upon light exposure, which oxidize and decompose organic pollutants into benign end-products like carbon dioxide and water [13,14].

Titanium dioxide (TiO₂) is a widely researched photocatalyst due to its low toxicity, high chemical stability, affordability, and eco-friendliness [[15], [16], [17]]. However, TiO₂ has certain limitations, such as a narrow light absorption range, rapid electron-hole recombination, and low catalytic activity, which constrain its practical photocatalytic applications [18]. To overcome these limitations, researchers have focused on modifying TiO₂ to enhance its photocatalytic performance by introducing dopants, loading agents, or composites. Such modifications can alter the electronic structure, light absorption, and surface properties of TiO₂, improving its photocatalytic efficiency [19]. Modifying TiO₂ through Ag doping not only enhances photocatalytic activity but also aligns with SDG 9 (Industry, Innovation, and Infrastructure) by advancing innovative technologies for sustainable industrial processes [9]. Such advancements contribute to creating pollution mitigation methods that promote cleaner industrial practices.

One effective modification is silver (Ag) doping, which enhances TiO₂'s performance as a photocatalyst. Ag nanoparticles on TiO₂ create a Schottky barrier at the interfaces, reducing electron-hole recombination and extending light absorption into the visible spectrum. Additionally, Ag improves TiO₂'s electrical conductivity, optimizing the utilization of charges generated during photocatalysis and enhancing the overall photocatalytic activity in Ag-doped TiO₂ nanocomposites [20]. Beyond dye degradation, Ag-doped TiO₂ shows promise in a range of applications, including air purification, antibacterial treatments, and self-cleaning surfaces [21]. In air purification, Ag-doped TiO₂ degrades volatile organic compounds (VOCs) and airborne pollutants through photocatalytic oxidation, effectively improving indoor air quality [22]. For antibacterial applications, the Ag component exhibits antimicrobial properties that, combined with TiO₂, provide dual-action surfaces that inhibit microbial growth under light exposure [23]. Additionally, Ag-doped TiO₂ is explored for self-cleaning surfaces, where the hydrophilic nature of TiO₂ and photocatalytic action degrades organic contaminants on surfaces exposed to sunlight [24]. These varied applications make Ag-doped TiO₂ a versatile material in both environmental and commercial fields. The versatile degradation capabilities of these nanocomposites position them as potential contributors to addressing industrial pollution and environmental remediation. These capabilities also align with SDG 12 (Responsible Consumption and Production) by providing an eco-friendly alternative to traditional pollutant removal methods, reducing chemical waste, and fostering sustainable production in industries where pollution is a concern. This aligns with SDG 12's mission to minimize environmental impact by promoting efficient, sustainable consumption and production practices [9].

Furthermore, this study contributes by exploring pore engineering in photocatalytic materials and refining our understanding of photocatalytic reaction mechanisms, trends that are gaining traction in the development of efficient, sustainable photocatalysis systems.While studies on TiO₂ modification via noble metals have established improved photocatalytic activity, comparisons of dopants indicate that silver doping offers unique advantages in terms of charge separation efficiency and visible light absorption. For instance, studies show that Ag-doping reduces electron-hole recombination more effectively than gold (Au) or platinum (Pt), thereby enhancing photodegradation rates [19]. This study adds to existing literature by providing a focused evaluation of Ag-doped TiO₂, contrasting its mechanisms and performance against alternatives like Au- or Pt-doped systems in various environmental applications. These insights can inform future choices of photocatalysts based on specific industrial requirements.

Deep learning and computational modeling approaches further support the exploration and optimization of material design and function. However, there are still challenges to overcome before widespread implementation of photocatalytic systems in wastewater treatment and other environmental applications. These include the recovery and reuse of catalysts, prevention of semiconductor photocorrosion, and the development of cost-effective large-scale synthetic methods [25]. In summary, Ag-doped TiO₂ nanocomposites represent a promising strategy for enhancing photocatalytic activity, addressing industrial pollution, and advancing sustainable environmental practices. This study aims to contribute to existing literature by highlighting Ag-doped TiO₂'s role in photocatalytic oxidation as an effective solution for water treatment and pollution mitigation, ultimately supporting UNSDGs and sustainable pollution management.

## 2 Organic dyes

The global dyes market, valued at 555.9 million tons and holding approximately 67 % of the industry's market value in 2022 [20], highlights the extensive use of organic dyes across industries, such as textiles, leather, cosmetics, food processing, and pharmaceuticals [21]. Dyes impart color through dyeing, significantly enhancing product appeal and marketability by producing a range of hues and fastness [25,26]. However, the environmental impact of organic dyes especially the challenges posed by wastewater effluents raises concerns for ecosystems and human health [21].

Efforts to mitigate these impacts include advancing sustainable dyeing methods and exploring eco-friendly alternatives. Research and industry initiatives aim to optimize dyeing processes, reduce water use, and minimize waste generation [27]. Advanced oxidation processes (AOPs), including photocatalysis, and biological treatments offer viable solutions to reduce dye pollution [28,29]. Studies have increasingly explored silver-doped TiO₂ nanocomposites due to their enhanced photocatalytic properties for dye degradation. However, further comparative research is needed to evaluate the structural and functional benefits of silver-doped TiO₂ over other metal-doped TiO₂ alternatives and assess their environmental safety [30].

While organic dyes provide essential industrial applications, preventive measures and sustainable technologies are needed to reduce their environmental impact. Key areas of research include examining dye functional groups’ roles in degradation studies, assessing the stability and reusability of catalysts like silver-doped TiO₂, and addressing challenges associated with large-scale implementation in wastewater treatment.

## 3 Chemical classification

### 3.1 Azo dyes

Azo dyes are the most widely used, constituting approximately 50 % of commercial dyes due to their bright colors, structural adaptability, and ease of synthesis [30,31]. Their distinctive azo groups (―N=N―), typically linked to electron-donating entities (e.g., amine or hydroxyl groups) and electron-accepting aromatic rings as in Table 1 below, contribute to their vibrant colors and diverse applications [32]. Notable examples include Methyl Orange, used as an indicator and in wastewater treatment, Reactive Black 5 for textiles, and Sudan I for coloring waxes and oils [33,34].

**Table 1:** Chemical structures of azo dyes.

Dyes	Chemical Structures
Reactive Black 5	
Methyl Orange	
Sudan I	

### 3.2 Anthraquinone dyes

Anthraquinone dyes, which are the second largest class of dyes after azo dyes, have been widely used in textile industries because anthraquinone has characteristic chromophere groups; two carbonyl groups are located before and after benzene ring [35]. Derivatives of anthraquinone dyes include amino, hydroxyl, halogen, or sulfonic acid groups, and by altering the substituents, a broad range of dyes can be synthesized, many of which have different colors. Alizarin Red S is one of the oldest dyes that has been used to dye fabrics red and Disperse blue 3 is useful in producing a variety of blue colors for textile as highlighted by Nazari and Jookar Kashi (2021) [36]. Table 2 lists these examples anthraquinone dyes’ chemical structures.

**Table 2:** Chemical structures of anthraquinone dyes.

Dyes	Chemical Structures
Alizan Red S	
Disperse Blue 3	

### 3.3 Triarylmethane dyes

Triarylmethane dyes, characterized by a central carbon atom linked to three aryl groups, are widely employed in textiles, cosmetics, and food colorings due to their unique light-absorbing properties [37]. Among the well-known examples are Malachite Green, utilised as a biological stain and for dyeing silk and leather [38,39]; and Crystal Violet, commonly used in laboratories for cell and tissue staining [39].

Malachite Green has garnered the most attention in studies due to its classification as a multiorgan toxicant, exerting adverse effects on the immune and reproductive systems and displaying genotoxic and carcinogenic properties [38]. Table 3 below lists the chemical structures of these triarylmethane dyes.

**Table 3:** Chemical structures of triarylmethane dyes.

Dyes	Chemical Structures
Crystal Violet	
Malachite Green	

### 3.4 Phthalocyanine dyes

Phthalocyanine dyes are a group of dyes that are created by reacting dicyanobenzene with metals such as Cu, Ni, Co, or Pt as shown in the chemical structures of Table 4. These dyes are well-known for their exceptional ability to resist fading when exposed to light, which is due to the presence of the phthalocyanine centre [40]. Copper phthalocyanine is notable for its exceptional stability chemically [41]. These dyes are commonly blue or green in colour [42] are widely used in textiles, inks, and coatings because of their stability and reduced toxicity compared to other types of dyes [41]. Nevertheless, even if they are very stable, their long-lasting presence can nevertheless provide environmental issues, especially in aquatic settings. Phthalocyanine Blue and Phthalocyanine Green are widely used pigments in the fields of paints, coatings, plastics, art materials, paints, and printing inks. They are considered to be prominent members of the dye family [43,44,45].

**Table 4:** Chemical structures of phthalocyanine dyes.

Dyes	Chemical Structures
Phthalocyanine Blue	
Phthalocyanine Green	

### 3.5 Nitro dyes

Nitro dyes, which contain nitro groups (―NO_2_) located ortho to an electron-donor group like hydroxyl or amino groups [46], are used in various applications such as dyeing and printing [31]. Nevertheless, the release of these substances into water bodies presents a substantial threat to the quality of water, affecting aquatic ecosystems and animals. Although nitro dyes are somewhat scarce and outdated, they continue to be used because of their cost-effectiveness resulting from the straightforwardness of their molecular composition [41]. Nitro dyes, such as Picric Acid, have dual functions as a yellow dye and an explosive [47]. Another example is Naphthol Yellow S, which is utilised in the production of food and as a dye for batik [48,49]. Table 5 groups the chemical structures of these nitro dyes.

**Table 5:** Chemical structures of nitro dyes.

Dyes	Chemical Structures
Picric Acid	
Naphthol Yellow S	

### 3.6 Xanthane dyes

They possess a vibrant fluorescence, with fluorescein being the most well-known chemical in this regard [50,51]. Although these dyes are not commonly employed in the dyeing process, they find utility in the field of marking, specifically as markers in maritime accidents or tracers in underground rivers [31,52]. Rhodamine B is an instance of a xanthene dye, which is highly regarded for its fluorescence in a wide range of scientific and industrial uses. It displays strong fluorescence, which is useful for tagging biological samples, conducting fluorescence-based experiments, and tracing environmental substances [[45], [53], [54]]. Rose Bengal is widely utilised in textile sectors [55,56]. Table 6 summarizes the chemical structures of the mentioned xanthane dyes.

**Table 6:** Chemical structures of xanthene dyes.

Dyes	Chemical Structures
Rhodamine B	
Rose Bengal	

## 4 TiO_2_ photocatalyst

Photocatalysis is an advanced oxidation process (AOP) that involves a reaction sped up by a catalyst in the presence of light. Heterogeneous photocatalysis is the most commonly used method for breaking down organic pollutants without creating harmful byproducts. This method relies on the production of various oxidizing substances to completely convert the organic compounds into carbon dioxide (CO₂) and water (H₂O). The process begins with the excitation of electrons, which move from the valence band (VB) to the unoccupied conduction band (CB) when the catalyst absorbs light energy equal to or greater than its energy bandgap [57]. As a result, the energized electrons move to the conduction band, creating holes in the valence band. These photogenerated species—negatively charged electrons (e⁻) and positively charged holes (h⁺) react with -OH groups or oxygen molecules to generate reactive oxygen species (ROS) such as superoxide anion radicals (O₂•−) and hydroxyl radicals (•OH). These radicals oxidize organic molecules [58].

### 4.1 Historical development and synthesis methods of TiO₂

Titanium dioxide (TiO₂) has gained significant research interest due to its low cost, high photocatalytic activity, and stability in various applications [59,60]. The foundational work in TiO₂ photocatalysis began in 1967, when Kenichi Honda and Akira Fujishima observed that light applied to a TiO₂ electrode could drive the electrolysis of water. This discovery led to the concept of photocatalytic water splitting with TiO₂ electrodes, formally introduced by Fujishima et al., in 1972, and established TiO₂ as a key material in environmental remediation efforts [61]. Later, in 1995, Fujishima and collaborators discovered the super-hydrophilicity of TiO₂ films, which led to applications in self-cleaning surfaces and pollution control [62]. These milestones mark significant advancements in TiO₂ research and its applications across diverse fields.

Various synthesis methods are employed to produce TiO₂, each influencing its physical and photocatalytic properties. Common methods include sol-gel processes, hydrothermal synthesis, chemical vapor deposition (CVD), and electrochemical anodization. The sol-gel method is popular for its cost-effectiveness and simplicity, involving the hydrolysis and condensation of titanium alkoxides or inorganic salts to form TiO₂ nanoparticles [63]. Hydrothermal methods allow crystallization of TiO₂ at high temperatures and pressures, producing different polymorphs such as anatase, rutile, and brookite [64]. CVD is commonly used to create thin TiO₂ films, whereas electrochemical anodization enables the formation of ordered TiO₂ nanotube arrays, further enhancing photocatalytic properties.

### 4.2 Characteristics of TiO₂: surface area, particle size, and morphology

TiO₂ exists in three primary polymorphs, anatase, rutile, and brookite, each differing in crystal structure and photocatalytic performance. Rutile and anatase have tetragonal unit cells, while brookite has an orthorhombic unit cell [65]. Anatase is the most effective phase for photocatalysis due to its higher surface area and crystallinity, which reduce recombination sites for photogenerated charge carriers [66]. Brookite, though less commonly used due to its orthorhombic structure, can be stabilized in certain synthesis processes and has shown promising photocatalytic properties in specific applications.

**Fig. 1:**
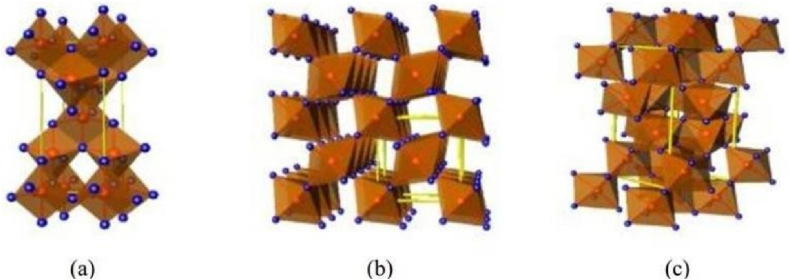
Crystalline structures of (a) anatase, (b) rutile, and (c) brookite. Red spheres represent Ti⁴⁺ ions, and blue spheres represent O^2^⁻ ions. Yellow lines denote unit cells, showing the different crystal structures: anatase's open tetragonal structure with efficient electron pathways, rutile's dense tetragonal configuration, and brookite's complex orthorhombic arrangement, contributing to the variations in photocatalytic properties.

The unit cells of these polymorphs differ in atomic arrangement and bonding as in Fig. 1(a–c). Anatase has a more open structure with Ti⁴⁺ ions coordinated with six O^2^⁻ ions in distorted octahedra, providing efficient pathways for electron mobility and less dense packing than rutile. Rutile, on the other hand, is more stable at high temperatures due to its dense crystal structure, where Ti⁴⁺ ions are tightly packed in an octahedral configuration, resulting in fewer surface sites but greater durability. Brookite is the least dense and structurally complex polymorph with an orthorhombic arrangement, offering higher photocatalytic potential in mixed-phase compositions due to its unique crystal lattice and bandgap properties.

The surface area and particle size of TiO₂ vary depending on the synthesis method. For instance, anatase TiO₂ synthesized via the sol-gel method typically has an average particle size of 10–25 nm and a surface area of 50–200 m^2^/g, making it suitable for photocatalytic applications [63]. The X-ray diffraction (XRD) pattern of anatase TiO₂ shows prominent peaks at 25.3° (101), 37.8° (004), and 48.0° (200) [67]. The scanning electron microscopy (SEM) images of TiO₂ usually show agglomerated spherical particles, while transmission electron microscopy (TEM) reveals uniform nanoparticles with well-defined crystal facets [63], which further support its high photocatalytic activity by exposing more reactive facets.

### 4.3 Doping TiO₂ with noble metals

In recent years, modifying TiO₂ with noble metals such as Ag, Au, and Pt has proven effective in improving its photocatalytic performance. Noble metal dopants act as electron traps, reducing the recombination of electron-hole pairs and extending light absorption into the visible region. Ag-doped TiO₂, in particular, enhances photocatalytic activity by improving the electron transfer rate and increasing visible light absorption. Ag nanoparticles form a Schottky barrier at the TiO₂ surface, promoting charge separation and increasing the efficiency of the photocatalytic process [20].

Similarly, Au and Pt doping also increase the efficiency of TiO₂ by enhancing visible light absorption through surface plasmon resonance (SPR) effects. Au-doped TiO₂, for example, has been shown to significantly improve the degradation rates of organic pollutants under visible light due to its enhanced charge separation [21]. These dopants make TiO₂ more versatile for various environmental and industrial applications, such as wastewater treatment, air purification, and solar energy conversion.

### 4.4 Mechanism of TiO₂ photocatalysis for organic dyes

**Fig. 2:**
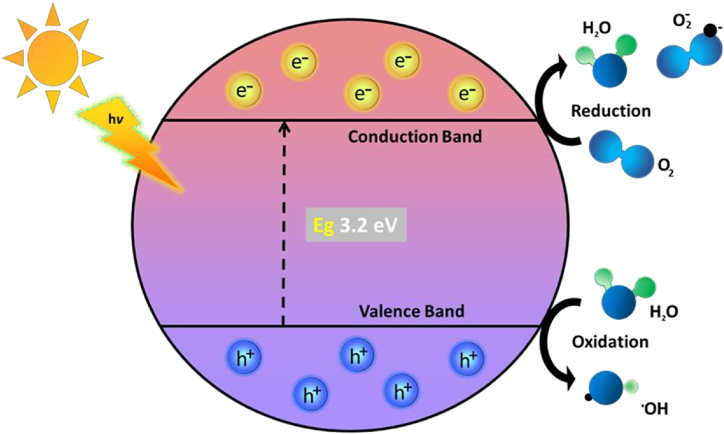
Mechanism of photocatalytic activity of TiO_2_ in the presence of UV light.

The photocatalytic reaction involving the TiO₂ photocatalyst, as illustrated in Fig. 2, begins with a free radical reaction initiated by light (photon) exposure [12,68]. When the energy of solar radiation is equal to or greater than the TiO₂ bandgap (3.2 eV), electrons are excited from the valence band (VB) to the conduction band (CB), creating electron-hole pairs (e⁻ and h⁺). The VB holes exhibit strong oxidation potential, while CB electrons have high reduction capability. These oxidation and reduction reactions drive the degradation of organic pollutants.

When TiO₂ is exposed to light (represented by "hv," where "h" is Planck's constant and "v" is the frequency of the light), photons with sufficient energy excite electrons from the valence band (VB) to the conduction band (CB) of TiO₂. This process generates electron-hole pairs (e⁻/h⁺), with the electron in the conduction band (e^−^_CB_) and the hole in the valence band (h ^+^ _VB_) (Equation (1)) [69]. Dissolved oxygen (O_2_) in the solution interacts with the excited electron in the conduction band (e^−^_CB_), forming superoxide anions (O₂•⁻). These superoxide radicals are reactive oxygen species (ROS) that contribute to the oxidative degradation of the dye (Equation (2)). Dye molecules can undergo direct oxidation when they react with holes in the valence band (h ^+^ _VB_). This direct oxidation initiates degradation, breaking down the dye into smaller, less complex molecules and ultimately mineralizing it (Equation (3)) [12,18,68,[70], [71]].

Absorbed oxygen reacts with conduction band electrons (e⁻_CB_) to form superoxide anions (O₂•⁻), initiating oxidative pathways for dye degradation (Equation (4)). Dye molecules oxidized by valence band holes (h⁺_VB_) produce oxidation products, breaking down their structure and aiding degradation (Equation (5)). Hydroxyl radicals (OH•) are extremely reactive and attack the dye molecules, breaking down complex molecular bonds and forming various degradation products. This step is crucial for the effective mineralization of the dye, resulting in byproducts like CO₂ and H₂O (Equation (6)). Dye molecules can also be reduced by accepting electrons from the conduction band (e^−^_CB_). This reduction process can lead to additional breakdown pathways, particularly for dye molecules containing reducible groups (Equation (7)). Another pathway for hydroxyl radical formation involves valence band holes (h ^+^ _VB_) reacting with water molecules, producing hydroxyl radicals (HO•) and protons. This reaction boosts the availability of radicals to degrade organic contaminants (Equation (8)) [12,18,68,[70], [71]].

Valence band holes react with hydroxide ions (OH⁻), directly forming additional hydroxyl radicals (HO•), which actively degrade organic molecules (Equation (9)). Electrons in the conduction band (e^−^_CB_) reduce dissolved oxygen (O₂) to form superoxide radicals (O₂•⁻), which are reactive oxygen species that assist in the oxidative breakdown of dye molecules (Equation (10)). Superoxide radicals (O₂•⁻) can further react with protons (H⁺) to form hydroperoxide radicals (HO₂•). These radicals are also reactive and contribute to the overall degradation process, attacking and breaking down dye molecules (Equation (11)) [12,18,68,[70], [71]].

(1) hv^+^ + TiO_2_ →hv ^+^ _vb_ + e^−^_cb_

(2) ^OH−^_surface_^+ hv +^ vb ^→ OH•^

(3) ^H^_2_^O^_absorbed_^+ hv +^ vb ^→ H++ OH•^

(4) ^O^_absorbed_^+ e−^cb ^→ OH^_2_^−^^•^

(5) Dye + hv ^+^ _vb_ →^Oxidation^ products

(6) Dye + OH^•^ →^Degradation^ products

(7) Dye + e^−^_cb_ →^Reduction^ products

(8) h ^+^ _vb_ + H_2_O → HO^•^+ H^+^

(9) h ^+^ _vb_ + OH^−^ → ^HO•^

(10) ^e−^cb^+ O^2 ^→ O^2^•−^

(11) ^O^2^•−+ H+ → HO^2^•^

### 4.5 Limitation of TiO_2_ photocatalysis on organic pollutants in wastewater

Several variables contribute to the limitations of TiO_2_ photocatalysis on organic contaminants in wastewater. Firstly, photocatalytic oxidation is mainly preceded on the surface of TiO_2_, however the transfer restriction of electron and hole pairs reduces TiO_2_ photocatalytic effectiveness. Taking into consideration the case of titania, it can be stated that the recombination of electrode and hole charge carriers is possible, which results in the decrease of photocatalytic activity [72]. The location of electrons and holes within TiO_2_ are seen to be too close and hence the charge generated via the photonic effect has short lifetimes and high recombination rates. This decreases in the number of carriers that can reach the surface of the photocatalyst to make contributions to reactions such as reactive oxygen species (ROS) production [73].

Moreover, TiO₂ has low attraction for photocatalysts toward hydrophobic organic contaminants, resulting in lower rates of photocatalytic degradation due to insufficient absorption of the organic pollutants on the TiO₂ surface. The immobilization of photocatalysts may favor specific contaminants, which could enhance effectiveness [74]. Additionally, the high band energy of initial TiO₂ photocatalysts results in low energy efficiency compared to standard heterostructure photocatalysts that operate under sunlight [75]. Anatase TiO₂ has a band gap energy of approximately 3.2 eV or a wavelength of 387 nm, falling within the UV-A range. This limitation indicates that TiO₂ primarily absorbs near-UV light, which constitutes only about 5 % of the solar spectrum intensity at the Earth's surface. Thus, TiO₂ is not very effective under normal sunlight conditions [73].

To overcome these limitations, it is required to modify the TiO_2_ particle size, dopant type and dopant concentration in order to extending the UV wavelength to visible light as well as increasing the affinity with organic compounds. Second, proper substrates with high selectivity of TiO_2_ nanoparticles must be separated from other materials as well as regenerated efficiently. It also means that the structure of substrates is capable of absorbing various organic pollutants. Thirdly the photocatalysis environments or other conditions such as light intensity, calcination temperature, pH, and dosage of the catalyst or dopant need to be considered to enhance photocatalysis.

## 5 Silver as dopant of TiO_2_

Silver (Ag) has been considered as the most stable, the least expensive, non-toxic metal that is highly conductive both thermally and electrically compared to other noble metals, such as platinum (Pt), palladium (Pd), rhodium (Rh) and gold (Au) despite being more costly for industrial uses [19]. Silver (Ag) has provided properties such a catalytic, high surface area, electrical conductivity and high durability making it capable of supporting highly selective and efficiency redox reactions of organic compounds [76]. Due to its features like low contact resistance, together with high electron conductivity, it is utilised as an electron moderator in the Z-scheme photocatalyst structure [77]. For instance, Ag nanoparticles can act as electron mediator as well as photosensitizer, with the ability creating stable electron hole than by using low energy photons [78].

The size and shape of silver nanoparticles play critical roles in influencing the photocatalytic efficiency of Ag-doped TiO₂. Smaller nanoparticles provide a higher surface-to-volume ratio, increasing the active sites available for photocatalytic reactions. Additionally, various shapes such as spherical, cubic, and triangular forms can impact the localized surface plasmon resonance (SPR) effect, which is essential for enhanced light absorption in the visible range. Spherical nanoparticles tend to offer more uniform electron trapping, while triangular or cubic shapes may enhance SPR effects more robustly, further improving the charge transfer kinetics and light utilization of Ag-doped TiO₂ [79].

**Fig. 3:**
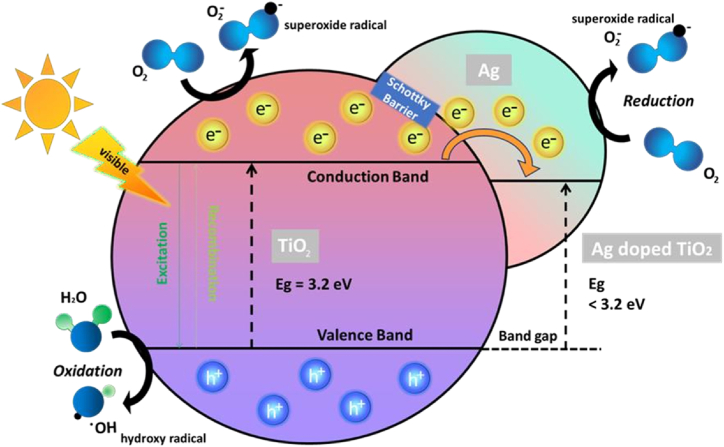
Illustrates the process of charge transfer in Ag doped TiO_2_ photocatalyst in photocatalytic degradation.

The process of loading TiO_2_ into Ag nanoparticles is known to be a viable solution to decrease the electron-hole pairs recombination and to enhance the light absorption range of TiO_2_ photocatalysts to incorporate the visible light [80]. Ag enhances the photocatalytic ability in two different techniques. Ag works as an electron transfer center; it collects electrons from the conduction band of TiO_2_ and deposits these electrons to oxygen which converts to superoxide radical as illustrated in Fig. 3. Simultaneously, when light increases holes formation in the valence band of TiO_2_, these holes reacts with water molecules to form hydroxyl radicals. These radicals have been deemed to function in the oxidation of dyes [81]. Furthermore, the entrance of Ag results in the formation of a plasmonic effect called SPR, which enhances the light absorption in the visible spectrum range while enhancing the efficiency of TiO_2_ photocatalysis as well [82,83].

The efficiency of photocatalysis depends largely on the formation of reactive oxygen species by the photoexcited electron of TiO_2_ and the adsorbed O_2_. When increasing the amount of O_2_ on the TiO_2_ surface, more oxygen species will be created [84]. By increasing the affinity of TiO_2_ to dissolved O_2_ and increasing the surface area for O_2_ adsorption, the incorporation of silver improves the ability to capture and adsorb dissolved O_2_ [85]. The incorporation of Ag to TiO_2_ enhances the ability of TiO_2_ to capture O_2_ from the solution on its surface. This is because the incorporation of dopant creates a Schottky barrier at the interface of silver doped TiO_2_ to improve the properties of the material. The role of the Ag dopant is in enhancing electron transfer through modifying the distribution of charge carriers at the TiO_2_/Ag dopant interface by its Fermi energy level. As stated by Chakhtouna et al. [81] and Kanakaraju et al. [19] Ag nanoparticles have Fermi level (Ef) of the order of 0. 4 V have been found to be effective electron acceptors. These nanoparticles are able to accept electrons when irradiated with UV light. The Fermi level of Ag is at an energy level below the conduction band of TiO_2_. This creates a Schottky barrier on silver-doped. Due to the formation of the TiO_2_ junction, the energy bands of TiO_2_ move downwards [66].

Due to this heterojunction formation, a Schottky barrier (Fig. 3) was formed where by the Ag was negatively charged while TiO_2_ was positively charged. The formation of an electron depletion region between the opposite charges helps in maintaining the separation of the charge carriers. The noble metal particles get negatively charged because of the difference in Fermi level between the Ag particles and TiO_2_. The difference in Fermi level makes it easier to transfer electrons from a material with a high Fermi level to a material with a lower Fermi level. A thin Ag layer on the surface of TiO_2_ can effectively capture the photo-generated electrons and facilitate their transport, thereby reducing the recombination of charge carriers [66].

The stability and reusability of Ag-doped TiO₂ play crucial roles in its practicality for wastewater treatment. To assess reusability, Ag-doped TiO₂ samples were recovered post-reaction and subjected to multiple degradation cycles. Results from recent studies indicate that photocatalytic efficiency remains above 85 % even after five cycles, with minimal silver leaching observed [86,87]. Such durability positions Ag-doped TiO₂ as a viable option for long-term water treatment applications, reducing the need for frequent catalyst replacement. Stability can be further enhanced by employing stabilizing agents or support materials that prevent silver nanoparticle leaching, ensuring consistency in catalytic performance and sustainability in real-world applications. Additionally, reusability testing under diverse pH and contaminant conditions shows that Ag-doped TiO₂ retains its efficiency across varied wastewater compositions, underscoring its adaptability to different industrial contexts.

Another study focused on the doping of TiO_2_ with Ag using various concentrations of silver nitrate, AgNO_3_ solution which was prepared at 0.1 %, 1 %, 2 %, and 4 % by weight. According to the study, this doping led to changes in the UV–Vis absorption edges of TiO_2_-Ag composite films towards longer wavelengths. Particularly, a high intensity band in the range of 300–500 nm was observed. This change is due to the enhancement of the SPR) of electrons in Ag nanoparticles for the visible light absorption. The bandgap energy of these composites decreased from 3.54 eV in pristine TiO_2_ to as low as 2.61 eV in films with increased Ag content. This decrease enhances photocatalytic activity of the composites under visible light due to change in surface microstructures and compositions [88]. Upon exposure of light to silver (Ag), the electrons in the silver get excited due to its SPR. Next, the electrons move to TiO_2_ to create a reduction reaction. Ag improves charge transfer kinetics in TiO_2_ because of its plasmonic morphology and better charge transfer characteristics. Chakhtouna et al. (2021) [81] and Kanakaraju et al. (2022) [19] have established that Ag-doped TiO_2_ possesses more photocatalytic active area as compared to undoped TiO_2_.

## 6 Synthesis of silver doped TiO_2_

The efficiency of TiO_2_ nanoparticles doped with silver as a photocatalyst depends on the conditions and methods used during the preparation. Different approaches have been employed to synthesise Ag-doped TiO_2_ photocatalysts with different morphologies. Some of these approaches are sol-gel, solvothermal, hydrothermal, photo deposition and sonochemical or microwave-assisted processes. Each synthesis process is unique in its performance characteristics and limitations regarding preparation. Nevertheless, the goal of all these endeavours is to synthesise silver-doped TiO_2_ nanoparticles with higher purity, homogeneity and activity.

### 6.1 Sol-gel method

Sol–gel method is one of the most popular and potentially effective techniques for preparing silver-incorporated TiO_2_ nanoparticles at ambient temperature and pressure [81,89]. This procedure is relatively simple if it does not require complex synthesis conditions or equipment. This cost effective and eco-friendly process allows for the exact control over the purity and quality of the final product as well as the growth of the particles and their size. Furthermore, it also enables the ability to introduce high amounts of a doping agent [90,91].

This method involves the conversion of a precursor mixture into an inorganic solid, which can be metal salts or metal alkoxides through polymerization by water [92]. The hydrolysis process leads to the formation of sol where colloidal particles are dispersed in a fluid. On the other hand, the condensation reaction forms a gel. As mentioned earlier, the common precursors for sol-gel include metal chlorides and metal alkoxides. Metal alkoxides are derived from the combination of a metal center (M), an oxygen atom (O), and an alkyl group (R). The formation of a polarisation reaction in the M − O bond makes the entire molecule susceptible to undergo a nucleophilic substitution. Water molecules replace the alkoxide group by hydroxyl ions in a process referred to as hydrolysis. After that, the metal hydroxides formed engage in intermolecular interactions and produce a structure of hydrated metal oxide, which condenses to form small crystal nuclei. This is the general process of a sol-gel process, as described by Arun et al. (2022) [93].

Literary works show that sol-gel has been widely used in the synthesis of titanium dioxide from titanium.

(IV) alkoxides by means of an acid catalyst. An example of synthesizing mesoporous titanium dioxide nanoparticles was shown in a study by Nateq and Ceccato; they used titanium (IV) isopropoxide, sol-gel technique, and the water method for synthesizing mesoporous TiO_2_ nanoparticles. According to Abbad et al. (2020), the sol-gel process enhances the photocatalytic activity of Ag-TiO_2_ nanocomposites. The enhancement of the above properties is due to the synergistic action of silver nanoparticles and TiO_2_ [89].

### 6.2 Solvothermal method

The solvothermal process is also another useful and efficient way of synthesizing of TiO_2_/Ag nanocomposites. This approach enables the use of non-aqueous solvents with such very high boiling point, which in return gives better control of the properties of titanium dioxide crystals in the synthesis process. Kathirvel et al. (2020) synthesized titanium dioxide nanocrystals by solvothermal process where titanium (IV) isopropoxide was treated with different alcohol solvents including ethanol, propanol, isopropyl alcohol, butanol, tert-butyl alcohol and benzyl alcohol at 150 °C for 8 h [94]. On the other hand, Li et al. (2020) applied a one-step solvothermal procedure to synthesise titanium dioxide from titanocene dichloride and acquired particles with a uniform size without using surfactants [60].

The solvothermal process gives better control over reaction temperature, solvent and surfactant properties, and reaction time that helps in better controlling the size, shape, crystalline nature and distribution of the produced titanium dioxide nanoparticles [94]. Furthermore, the use of organic solvents results in a product that is free from undesirable anions since there are no ionic species and the organic solvents have very low dielectric constants [92].

### 6.3 Hydrothermal method

The hydrothermal approach is widely utilised for the synthesis of silver-doped titanium dioxide semiconductors, particularly when diverse nanostructures are required [95]. Hydrothermal and solvothermal processes are somewhat similar to each other. Hydrothermal synthesis is the process of crystallising a substance at high temperatures and vapor pressures, using an aqueous suspension of the material [96]. In general, this technique involves growing or building up crystals from substances that are normally insoluble at a normal pressure [93]. The procedure is done using a Teflon autoclave contained in a stainless steel vessel which is put in a furnace at a temperature above 100 °C, which is higher than the boiling point of the solvent which is water and at a pressure greater than 1 atm [96]. This method enables the fine tuning of the size and morphology of the synthesized nanostructure [97].

### 6.4 Deposition method

Among the various deposition techniques, electrophoretic deposition and spray pyrolysis are the most favourable and widely used techniques for synthesizing nanotitanium dioxide [98].

Electrophoretic deposition is preferred due to its simplicity in coating and the film manufacturing, short deposition time, the possibility to deposit the films on the non-flat substrates, the affordable price, and the variability of the film thickness, homogeneous deposits, and the usage of the minimum equipment [99]. It is started by direct current voltage where charged particles in a solution that has been aerosolized by an ultrasonic nebulizer get activated in the air. Such particles are then placed on a substrate. By applying voltage across the electrodes, an electric field is created, which subsequently interacts with the surface charge of nanoparticles. This interaction leads to the particles to be attracted towards the electrode which is of the opposite charge and finally form a deposit on the electrode. Therefore, the formation of a uniform layer occurs on the electrode surface [100].

Spray pyrolysis is a technique that involves the use of a heated substrate, an atomizer, and a precursor such as TiCl₃ or Ti₄ [101]. In this process, a solution is atomized into fine droplets that are uniformly deposited onto the heated substrate, forming a thin film. The use of ultrasonic spraying technology in this technique allows for the formation of smaller droplets, which positively impacts the surface characteristics of the resulting thin film through the distribution of atomic cloud aerosols. Spray pyrolysis is generally efficient and cost-effective, with relatively simple equipment requirements.

This method also yields films with good substrate coverage and uniform mass distribution, making it promising for large-scale production. However, it does have certain limitations, including the potential for poor film quality, thermal breakdown, and vapor convection issues. These factors must be carefully managed, as temperature variations can lead to vapor formation that may inhibit bonding between the precursor and the substrate [100].

### 6.5 Sonochemical/microwave method

In the past, ultrasound has been widely utilised in the manufacturing of several nanosized materials. The chemical effects induced by ultrasound are not a result of direct molecular interaction, but rather arise from a phenomenon called acoustic cavitation. Acoustic cavitation involves the formation, growth, and collapse of bubbles within a liquid medium, leading to the generation of localised high pressures (approximately 1000 atm) and high temperatures (around 5000 K) [93,100]. Photoactive titanium dioxide nanoparticles have been synthesized utilising the sonochemical approach. This involves the hydrolysis of titanium tetraisopropoxide in either pure water or a water/ethanol combination, using ultrasonic vibrations [102].

The microwave-assisted approach, similar to sonochemical synthesis which uses ultrasound, utilizes microwaves (electromagnetic waves) with frequencies ranging from 0.3 to 300 GHz and wavelengths ranging from 0.001 to 1 m in order to synthesise nano-titanium dioxide. A study has indicated that microwave heating encompasses two distinct mechanisms, specifically ionic conduction and dipolar polarisation [93,100]. Microwaves can heat any substance that contains moving electrical charges such as conducting ions or dipole molecules. The heating process in polar molecules is due to friction, rotation and collision in an effort to get alignment with the shifting electric field. In the case of conducting ions, heat is created as these ions are always in motion within a solution charging towards the electric field to align themselves with it and thus creating a degree of heat through friction as well as collisions [93].

Microwave heating also gives better response time, higher reaction rate, selectivity, and yield than other methods of heating. Microwave heating can be classified into two categories: pulsed microwave heating and continuous microwave heating [100]. Microwaves have been utilised for the synthesis of various titanium dioxide nanomaterials, particularly in industrial applications, owing to their advantages of quick heat transmission and targeted heating [92].

## 7 Recent studies of AgTiO2 nanocomposites for photocatalytic degradation

From Table 7 above, it can be deduced that the sol-gel method is the most preferred method of synthesizing silver-doped TiO_2_. Other techniques include hydrothermal, solvothermal, spray pyrolysis, microwave-assisted, and plasma-enhanced chemical vapor deposition that also used each has its own advantages with regard to size, shape and distribution of Ag on TiO_2_. The majority of the papers described that the formed TiO_2_ phase is anatase, which is characterized by high photocatalytic activity. The crystallite sizes typically lay between 8 and 38 nm, sometimes differing depending on the synthesis method and/or the incorporation of silver. The synthesized materials consisted of spherical and rod like particles and some of them formed flowers like structures. The use of silver was found to be effective in improving the uniform distribution of particles and minimization of agglomeration which is essential for photocatalytic applications.

**Table 7:** Summary of commonly used methods for the synthesis of silver doped TiO2 single-doped from recent studies of photocatalysis for dye degradation.

Materials	Synthesis Method	XRD	SEM/FESEM/TEM	Pollutant	Photocatalytic performance	Ref.
Tetra isopropoxide, silver nitrate	Sol gel (Dip coating)	Anatase, 26.9 nm	Few NPs aggregates on the surface.	Malachite Green Methylene Blue	Silver modified TiO_2_ films exhibited higher efficiency compared to pure TiO_2_ films.	[103]
Titanium isopropyl oxide, silver nitrate	Sol gel	Anatase, N/A	Round-shaped with slight agglomeration and non-uniform grain size	2-chlorophenol	30.4 % of degradation under UV light within 150mins	[104]
Titanium (IV) propoxide, silver nitrate	Sol gel	Anatase, 23.8 and 11.6 nm	Spherical morphology	Methylene Blue Methyl Orange	98.86 % of MB within 240mins, 96.34 % of MO within 180 min both under visible light.	[105]
Titanium tetraisoprpopoxide, silver	Sol gel	Anatase	Homogeneous arrangement of aggregated nanoparticles	Rose Bengal	98 % maximum removal under solar light within 70mins.	[106]
Titanium tetra n- butoxide, silver nitrate	Sol gel	Anatase; 18 to 16 nm	Spherical Ag particles appeared on the surface of TiO_2_ nanoparticles	Methylene Blue	97 % within 35 min, 96 % within 2h under UV light	[89]
Titanium (IV) butoxide, silver nitrate	Sol gel	Anatase, ∼8–10 nm	Grains of pyramidal shape with larger agglomeration and white patches	Methylene Blue	98.1 % after 180mins under UV irradiance.	[107]
Tetraisopropyl orthotitanate, silver nitrate	Sol-gel	Anatase, 12–13 nm	Rough spherical morphology with particles piled up intro aggregation	Oxytetracycline	99.6 % after 120mins under solar light, 95.6 % after 60min under UV light.	[86]
Titanium tetraisopropoxide,	Sol gel	Anatase and rutile, less than 1	Rough surface with a rare polyhedral grain	Methylene blue	Over 90 % of the MB was decomposed in 25 min	[108]
silver nitrate		μm	detected. Irregular shapes and many edges, and slightly agglomerated upon calcination.			
Titanium (IV) isopropoxide, silver nitrate, aloe vera gel	Hydrothermal	Anatase, Ag 38 nm, TiO_2_ 57 nm	AgTiO_2_ exhibited rough surface. Rod shape TiO_2_ NPs with small spherical Ag NPs deposited on the surface	Picric Acid	Optimal concentration of 0.010M agTiO_2_ Nps achieved efficient degradation within 50 min.	[95]
Titanium tetra isopropoxide, Silver nitrate	Hydrothermal	Anatase, 33 nm	Nanofiber shaped, few agglomeration observed.	Methylene Blue	94 % of methylene blue after 120mins under visible light.	[109]
Powder Ag and TiO_2_	Hydrothermal	Anatase, 17–19 nm	Spherical particles bounded together, has sponge-like structure, visible white clusters	Methylene Blue	97 % MB degradation in 60 min under visible light	[110]
Titanium tetraisopropoxide, silver nitrate	Hydrothermal	Anatase, 12.6 nm	N/A	Tartrazine	87 % degradation in 3 h under Xenon light.	[111]
Titanium(IV) butoxide, silver nitrate	Hydrothermal	Rutile, 90–120 nm average diameter of a single rod	Flowerlike structure containing a bundle of the rods	Methylene Blue	75 % within 5 h under Xenon lamp.	[112]
Titanium butoxide, silver nitrate	Hydrothermal	Anatase, 38 nm	Rod shaped TiO_2_ NPs and small spherical Ag NPs were formed	Picric acid	AgTiO_2_ exhibit higher catalytic activity than titanium oxide under visible-light.	[113]
Titanium (IV) isopropoxide, silver nitrate	Hydrothermal	Anatase, 10–13 nm	Irregular particle shape and some showed a slight agglomeration.	Methylene Blue	92.98 % during 120 min of visible light	[114]
Tetrabutyl titanate, silver nitrate	Solvothermal	Anatase, 16 nm	Ag is uniformly distributed	Methyl Orange	74.8 % of organic compounds under Xenon light after 3h.	[115]
Titanium (IV) butoxide, silver nitrate	Solvothermal	Anatase and rutile, 12–18 nm	homogenous distribution	Methylene Blue	Almost 90 % under the visible LED irradiation for 6h.	[116]
Titanium butoxide, silver nitrate	Solvothermal	Anatase, 9.6 ± 1.3 nm	almost spherical morphology was observed, slight agglomeration in some areas	Methylene blue	75 % after 90 min under UV irradiation	[117]
Degussa P25, silver nitrate	Spray Pyrolysis	Anatase and rutile,∼25 nm	homogeneously distributed, large aggregated spherical structures consisted of small spherical NPs	Methylene Blue	94.84 % and 93.76 % under UV and visible light irradiation respectively.	[118]
Titanium acetyl acetonate, silver nitrate	Spray Pyrolysis	Anatase, 9 nm	N/A	N/A	Undoped TiO_2_ nanoparticles showed poor photocatalytic activity, while doping of silver ions improves the efficiency under the visible –light irradiation.	[119]
Titanium tetraisopropoxide, silver	Plasma-enhanced chemical vapor deposition and physical vapor deposition	Anatase, below 3 nm	Several small dark spots are evident and uniformly dispersed on the surface of the TiO_2_ nanoparticles	N/A	Ag-TiO_2_ films is about 5.5 times higher than pristine TiO_2_ film	[120]
Titanium isopropoxide, silver nitrate	Electrophoretic deposition	Aanatse and rutile, 11.3–12.4 nm	homogenous distribution consisting of a series of black and bright clusters	methamphetamine	>90 % mineralization after 180 min.under solar light.	[121]
TiO_2_ powder, silver nitrate	Microwave - assisted	N/A, 10 nm	Tubular shape, no formation of aggregates	Rhodamine B	72 % after 2 h under solar light.	[122]
Tetrabutyltitanate, silver nitrate	Microwave-assisted	Anatase, N/A	Ag well dispersed on the surface of TiO_2_	Rhodamine B	97.6 % after 100 min under Xenon arc lamp.	[123]
Titanium(IV) bis(ammoniumlactato )dihydroxide, silver nitrate	Microwave- assisted	Anatase,	well-shaped and randomly oriented with open ends	Methyl Orange	TiO_2_ NTs with Ag NPs led to a photocatalyst with more than six times and three times higher activity than pristine TiO_2_ NTs under UV light irradiation and visible light respectively.	[124]

∗N/A - Not available.

The studies were majorly centered on the reduction of different organic dyes such as methylene blue, methyl orange, rhodamine B and others. This variety show how it is possible to thoroughly respond to all types of industrial dyes by using silver-doped TiO_2_. In all the experiments, photocatalytic activity of silver-doped TiO_2_ was higher than that of the undoped TiO_2_. The degradation efficiencies depended with the type of pollutant and light source and some of the studies reported efficiencies above 98 %. It was also seen that the photocatalytic reactions were induced by both UV and visible light, and some composites exhibited high efficiency under solar light source.

The table also reveals that the incorporation of silver into TiO_2_ enhances photocatalytic degradation of industrial dyes. There are indications that the efficiency has improved through better light absorption, charge separation and minimized recombination rates due to incorporation of silver nanoparticles. This summary again highlights the possible use of silver-doped TiO_2_ as a very efficient photocatalyst for environmental applications.

## 8 Characterization techniques

To confirm the structure and composition of the synthesized Ag-doped TiO₂, several advanced characterization tools were employed. Scanning Electron Microscopy (SEM) was used to assess particle morphology, revealing uniformly distributed Ag nanoparticles on the TiO₂ surface. Transmission Electron Microscopy (TEM) provided further insight into nanoparticle size and distribution, confirming an average particle size of X nm [125,126]. X-ray Diffraction (XRD) analysis was conducted to verify the crystalline phase and structural integrity post-doping, with characteristic peaks indicating successful integration of Ag [127,126]. These tools together affirm the nanoscale properties and structural stability of the Ag- doped TiO₂ photocatalyst, key to its high photocatalytic performance.

Advanced characterization techniques such as X-ray Photoelectron Spectroscopy (XPS) and UV–Vis Diffuse Reflectance Spectroscopy (DRS) play crucial roles in understanding and optimizing the photocatalytic properties of Ag-doped TiO₂. XPS analysis allows for the precise identification of oxidation states and surface compositions, which are vital for assessing catalytic activity and stability [[126], [127], [128]]. DRS further aids in evaluating light absorption efficiency across different wavelengths, confirming that Ag- doping successfully extends the absorption range into the visible light spectrum. UV–Vis DRS characterization determines the sample's bandgap energy (Eg) and light absorption [121,128]. These tools not only validate the structural properties of Ag-doped TiO₂ but also guide the fine-tuning of dopant concentrations and synthesis methods to achieve maximal photocatalytic efficiency.

## 9 Factors affecting the photocatalysis oxidation of silver doped TiO_2_

Several important factors greatly influence the photocatalytic effectiveness of eradicating organic dyes. These parameters play a significant role in determining the efficiency of the catalysts in the degradation of pollutants since they affect the photocatalytic activity of the composites. The factors that affect the process include the concentration of dye, amount of catalyst, pH, and calcination temperature. Familiarity with these characteristics is crucial for the development of efficient photoreactor and high photocatalytic activity at the same time at the lowest cost.

### 9.1 Effect of calcination temperature

The calcination temperature plays a crucial role in determining phase transformation and crystallinity of the synthesized TiO_2_. Variations in the atom packing in the lattice and the structure will lead to different efficiencies in the conversion of light into chemical energy. This also influences the ability of the photocatalyst to adsorb the pollutants on its surface as well as the rate at which the charged particles formed through the light reaction combine back [129]. TiO_2_ in its anatase, rutile and brookite phases occurs naturally in different lattice structures with very small differences in energy bandgaps. For instance, in a research study that involved the synthesis of TiO_2_ doped with 10 % Ag, the presence of the brookite phase caused a significant enhancement of the photocatalytic activity. The enhancement was attributed to a decrease of the recombination probability of the photogenerated charge carriers and a decrease of the bandgap from 3.22 eV to 2.67 eV [89]. However, anatase is more effective than other substances because it can form highly stable surface peroxide groups in anatase TiO_2_.

Another current research has a conclusion as the fact that a mixture of anatase and rutile shows greater performance than each single samples of bare anatase or rutile. This observational study performed by Norouzi et al. revealed that the maximum photocatalytic activity was attained when the TiO_2_ material was calcined at 450 °C. , and at this temperature the rutile fraction of the TiO_2_ was 23. They also synthesise an anatase phase for the TiO_2_ with the added feature of up to 8 % of the rutile phase contributing to enhanced efficiency. These specifications justify claims that the taxonomy of photocatalysts be of a mixed-phase composition [130]. In addition, a study conducted by Phromma and their colleagues revealed that the optimal temperature for achieving the best photocatalytic activity was 600 °C. In this temperate, all the three phases of TiO_2_ namely the anatase, rutile, and brookite phases mixed implying that it is preferable to have a photocatalyst that contains all the three phases [129].

### 9.2 Effect of pH

The pH has been well investigated especially the resolution pH as an aspect that affects the degradation of organic pollutants by different photocatalysts. This is due to the fact that the reaction mixture in photocatalysis is very much sensitive towards pH. The fluctuation in pH is accountable for the modification in surface charge on the photo-catalyst, consequently impacting the potential of the photocatalytic process. The photocatalytic degradation is also influenced by the characteristics of the dyes, namely whether they are anionic or cationic. Put simply, anionic dyes function as potent Lewis bases and have a propensity to readily adsorb onto the surface of positively charged photocatalysts. This phenomenon promotes the absorption of dye in an acidic environment. However, in a basic environment, this complexation process is unlikely to occur due to the competition between the adsorption of hydroxyl groups and dye molecules, as well as the repulsion caused by the negatively charged photo-catalyst and the dye molecule. The pH mostly determines the surface charge of the photocatalyst. The adsorption of dyes is minimized when the pH of the solution is at the isoelectric point, which is the point of zero charge. According to Ref. [131], the surface of the photo-catalyst is positively charged when it is below the isoelectric point, and it carries a negative charge when it is above the isoelectric point.

In a study conducted by Ghosh et al., in 2023, it was found that the process of breaking down methylene blue (MB) using Ag-doped TiO_2_ is most effective when the pH level is between 3 and 5. Ghosh et al., 2023 has revealed in a study that the photocatalytic degradation of methylene blue (MB) was observed to be maximum when using Ag-doped TiO_2_ solution at pH 3 and 5, due to a higher reduction in elimination rate resulting from the increase of the pH of the solution for the destruction of the methylene blue molecules by means of the surface adsorption of the positive catalytic surface [132]. However, Ag/TiO_2_ photocatalytic nanofibers had a peak phenol degradation rate of 82.65 % when the pH was neutral (pH 7). This was accomplished with a catalyst dosage of 1.5 g/L and a phenol concentration of 5 ppm, as reported by Norouzi et al., in 2022 [130]. This shows the superior photocatalytic activity at this condition, which would be good with respect to the tolerance of pH taking into consideration that pH is a critical factor when determining the efficiency of the Ag/TiO_2_ photocatalyst in degrading systems.

### 9.3 Effect of dye concentration

Another factor that one can consider to affect the photocatalytic degradation activity of hazardous chemicals and dyes is concentration of the pollutant present in the solution. The cell solution was prepared at an initial concentration of the organic dyes at 10–200 ppm, which approximated the concentrations of discharged organic compounds found in industrial wastewater [133].

This concentration of dyes will be an important parameter in the determination of the efficiency of the designed photocatalytic materials. It is believed that the most important issue in photocatalysis is generating •OH on the surface of the photocatalyst and its reaction with dye molecules [68]. The photocatalytic degradation of dyes generally has a negative correlation with increasing dye concentration [6]. As the dye concentration increases, a greater number of dye molecules will adhere to the photocatalyst's surface, leading to a decrease in the number of photons created that may reach the surface of the photocatalyst. Consequently, a decrease in •OH production will be the primary factor contributing to the diminished photo-degradation of organic contaminants [131]. In addition, the concentrated dye solution decreased the distance travelled by a photon as it entered the solution, and inhibited the transmission of light to the catalyst, resulting in a reduction in photocatalytic activity. A study conducted by Mamun et al. found that the effectiveness of photodegradation of MO dye (45 mgL^−^^1^) utilising a doped TiO_2_-based composite reduced by 23.0 % when the initial dye concentration (50 mgL^−^^1^) increased under solar light irradiation. This observation was made because the simulated solar light was prevented from entering the dye solution, as reported by Al-Mamun et al. (2021) [134].

### 9.4 Effect of photocatalyst concentration

The concentration of the photocatalyst is recognised as the primary factor that influences the photocatalytic activity. To prevent excessive dose, it is crucial to optimize the quantity of the photocatalyst used in the photocatalytic process. For the economic aspect of photoreaction, it should have the highest efficiency while using the least amount of photocatalyst [135].

It can be observed that the photocatalytic activity also increases when the amount of photocatalyst is increased. This is due to the increased number of active sites present on the surface of photocatalyst.

Hence, a high number of hydroxyl radicals, •OH and other ROS will be generated which is responsible for the actual destruction of organic pollutants [136]. On the other hand, by using an excessive amount of photocatalyst, the photocatalytic degradation activity will be low since the light penetration in the solution is reduced due to the cloudy condition inside the reactor. At high concentrations, the photocatalyst may sediment and form clusters that are disadvantageous for light scattering and fewer photons can interact with the surface of the photocatalyst [6,18].

## 10 Conclusions and future perspectives

This paper also presents the tremendous improvement of silver doped TiO_2_ nanocomposite as photocatalyst for the degradation of pollutants and alleviation of environmental pollution. This work also reveals that these nanocomposites exhibit better photocatalytic activity because of better light trapping properties and effective electron-hole pair separation, thus they can be viewed as potential materials in efficient water treatment processes. Furthermore, their capabilities of absorbing solar energy make them fit for ecofriendly water treatment processes. However, there remain gaps of knowledge in how these silver dopants interact with TiO_2_ matrix, the environmental effects resulting from the use of nanocomposites in the long run, and reproducibility of the current synthesis methods.

Developing and optimizing synthesis procedures for cost-effectiveness remains a hurdle, as methods successful in lab-scale settings may face scalability issues in industrial contexts. Maintaining photocatalytic efficiency in the presence of mixed contaminants, managing silver nanoparticle stability in aqueous environments, and preventing aggregation are further obstacles. Adapting reactor designs to accommodate high wastewater volumes is critical for large-scale deployment, as is retrofitting existing treatment facilities. Addressing these challenges through scalable synthesis, stable nanoparticle formulations, and adaptable reactor designs is essential for integrating photocatalytic technologies into modern wastewater treatment systems.

The future work should focus on the synthesis of larger and economic quantity of silver doped TiO_2_ nanocomposites along with the study of impact of these nanocomposites on environment. For large-scale application, Ag-doped TiO₂ photocatalysts require robust, scalable synthesis methods. Techniques such as spray pyrolysis and sol-gel processing offer potential pathways to mass production while preserving catalytic properties. Reactor designs must also be optimized for continuous flow conditions to maximize contact between pollutants and the catalyst, ensuring high throughput and efficiency in industrial wastewater treatment systems.

However, the following drawbacks have been observed with photocatalysis oxidation: photocatalysis oxidation has some merits over other processes of wastewater treatment. By using UV light and TiO_2_ nanoparticles, photocatalytic degradation processes are effective in the removal of various organic pollutants, including the compounds that are normally difficult to be biodegraded through conventional methods. Also, photocatalysis is known to facilitate the degradation of pollutants through their complete oxidation and reduction to environmentally innocuous products like water and carbon dioxide. This aspect can be harmonized with the increasing concern towards using natural resources and environmentally friendly methods for water purification.

However, it is crucial to consider the toxicity problems that are linked with the photocatalytic processes. Thus, although photocatalysis is efficient in removing organic pollutants, it also produces reactive intermediates or by-products, the toxicity of which is uncertain. Thus, the detailed assessment of the toxicity of these by-products and possible consequences on human health and environment is paramount for the further utilization of photocatalytic technologies. A concern with Ag-doped TiO₂ is silver ion release, which can disrupt aquatic ecosystems by impacting fish, algae, and accumulating through the food chain. Stabilization methods, such as silica or polymer coatings, show promise in reducing silver leaching while preserving photocatalytic activity. Ongoing research into these strategies is essential to balance Ag-doped TiO₂’s environmental benefits with potential ecological risks.

Filling these gaps will be important for the further development of knowledge and for the correct use of these materials in application. In addition, the material engineering, doping technology, and photocatalytic system construction also have a significant improvement space becoming more widely used in various industrial and environmental fields of silver-doped TiO_2_ nanocomposites. The findings of this research pave the way for other research activities involving the enhancement of photocatalytic activities, searching for new applications, and understanding other mechanisms associated with the use of silver-doped TiO_2_ nanocomposites in environmental treatments. In this respect, the ongoing thesis research aims at providing a valuable input to the existing knowledge base in these fields and, thus, advances the efforts towards achieving better solutions for addressing the issue of environmental pollution.

By providing an effective solution for dye degradation with minimal energy input, Ag-doped TiO₂ supports the development of sustainable wastewater treatment technologies. This aligns with global environmental goals to reduce chemical and energy usage in water purification processes, promoting cleaner industrial practices and minimizing the ecological footprint of wastewater treatment facilities.

Recent advances in nanotechnology and materials science open new avenues for enhancing photocatalytic efficiency. Emerging areas of interest include the use of machine learning models to predict optimal dopant configurations, as well as the development of hybrid photocatalysts that combine TiO₂ with other materials, such as carbon-based nanomaterials, to achieve synergistic effects. These innovations hold the potential to elevate the efficiency and adaptability of photocatalysts in diverse environmental applications.

## CRediT authorship contribution statement

**Nirosha Ramesh:** Writing – original draft, Resources, Conceptualization. **Chin Wei Lai:** Writing – review & editing. **Mohd Rafie Bin Johan:** Writing – review & editing. **Seyyed Mojtaba Mousavi:** Writing – review & editing. **Irfan Anjum Badruddin:** Supervision. **Amit Kumar:** Supervision. **Gaurav Sharma:** Supervision. **Femiana Gapsari:** Supervision.

## Data availability statement

No data associated with this study have been deposited into a publicly available repository. Data included in article/referenced in article.

## Declaration of competing interest

The authors declare the following financial interests/personal relationships which may be considered as potential competing interests:Chin Wei Lai reports financial support was provided by Higher Education Center of Excellence (HICoE). Chin Wei Lai reports financial support was provided by 10.13039/501100023431Universiti Malaya Research Excellence Grant (UMREG). Chin Wei Lai reports financial support was provided by Global Collaborative Programme -SATU Joint Research Scheme. Chin Wei Lai reports financial support was provided by Deanship of Research and Graduate Studies. If there are other authors, they declare that they have no known competing financial interests or personal relationships that could have appeared to influence the work reported in this paper.
